# Identification of new glutamate decarboxylases from *Streptomyces* for efficient production of γ-aminobutyric acid in engineered *Escherichia coli*

**DOI:** 10.1186/s13036-019-0154-7

**Published:** 2019-03-21

**Authors:** Haina Yuan, Hongbo Wang, Ozkan Fidan, Yong Qin, Gongnian Xiao, Jixun Zhan

**Affiliations:** 1 Department of Biological Engineering, Utah State University, 4105 Old Main Hill, Logan, UT 84322-4105 USA; 2 School of Biological and Chemical Engineering, Zhejiang Provincial Collaborative Innovation Center of Agricultural Biological Resources Biochemical Manufacturing, Zhejiang Provincial Key Lab for Chem&Bio Processing Technology of Farm Produces, Zhejiang University of Science and Technology, Hangzhou, 310023 Zhejiang China; 3 Hangzhou Viablife Biotech Co., Ltd., 1 Jingyi Road, Yuhang District, Hangzhou, 311113 Zhejiang China

**Keywords:** Glutamate decarboxylase, γ-Aminobutyric acid, *Streptomyces*, Heterologous expression, Whole-cell biotransformation

## Abstract

**Background:**

Gamma (γ)-Aminobutyric acid (GABA) as a bioactive compound is used extensively in functional foods, pharmaceuticals and agro-industry. It can be biosynthesized via decarboxylation of monosodium glutamate (MSG) or L-glutamic acid (L-Glu) by glutamate decarboxylase (GAD; EC4.1.1.15). GADs have been identified from a variety of microbial sources, such as *Escherichia coli* and lactic acid bacteria. However, no GADs from *Streptomyces* have been characterized. The present study is aimed to identify new GADs from *Streptomyces* strains and establish an efficient bioproduction platform for GABA in *E. coli* using these enzymes.

**Results:**

By sequencing and analyzing the genomes of three *Streptomyces* strains, three putative GADs were discovered, including StGAD from *Streptomyces toxytricini* NRRL 15443, SsGAD from *Streptomyces* sp*.* MJ654-NF4 and ScGAD from *Streptomyces chromofuscus* ATCC 49982. The corresponding genes were cloned from these strains and heterologously expressed in *E. coli* BL21(DE3). The purified GAD proteins showed a similar molecular mass to GadB from *E. coli* BL21(DE3). The optimal reaction temperature is 37 °C for all three enzymes, while the optimum pH values for StGAD, SsGAD and ScGAD are 5.2, 3.8 and 4.2, respectively. The kinetic parameters including *V*_*max*_, K_m_, *k*_*cat*_ and *k*_*cat*_/K_m_ values were investigated and calculated through in vitro reactions. SsGAD and ScGAD showed high biocatalytic efficiency with *k*_*cat*_/K_m_ values of 0.62 and 1.21 mM^− 1^·s^− 1^, respectively. In addition, engineered *E. coli* strains harboring StGAD, SsGAD and ScGAD were used as whole-cell biocatalysts for production of GABA from L-Glu. *E. coli*/SsGAD showed the highest capability of GABA production. The cells were repeatedly used for 10 times, with an accumulated yield of 2.771 kg/L and an average molar conversion rate of 67% within 20 h.

**Conclusions:**

Three new GADs have been functionally characterized from *Streptomyces*, among which two showed higher catalytic efficiency than previously reported GADs. Engineered *E. coli* harboring SsGAD provides a promising cost-effective bioconversion system for industrial production of GABA.

**Electronic supplementary material:**

The online version of this article (10.1186/s13036-019-0154-7) contains supplementary material, which is available to authorized users.

## Background

**Fig. 1:**
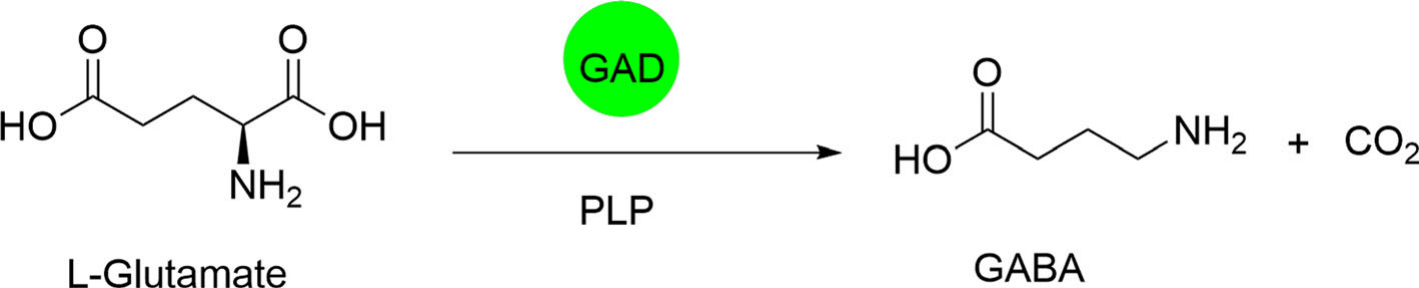
GABA production from L-glutamate by glutamate decarboxylase (GAD) with pyridoxal-5′-phosphate (PLP) as a cofactor

Gamma (γ)-Aminobutyric acid (GABA), also named 4-aminobutyric acid, is a non-proteinogenic water-soluble amino acid generally existing in various animal brains, plants and bacteria [1]. GABA considered as one of inhibitory neurotransmitters has been applied extensively to functional foods, pharmaceuticals and agro-industry due to its various biological activities such as hypotensive, epilepsy treatment, asthma control, sleep and memory improvement, hormone-regulating and obesity-preventing effects [2]. 2-Pyrrolidone was reported to be applied to chemically synthesize GABA, which composed the linear polymer compound named as nylon 4, a biodegradable plastic material [3]. As illustrated in Fig. 1, GABA is biosynthesized in the cells through decarboxylation of L-glutamate catalyzed by glutamate decarboxylase (GAD; EC4.1.1.15) [4, 5].

GAD is a pyridoxal 5′-phosphate (PLP)-dependent intracellular enzyme widely distributed in various biogenetic resources [6]. Most reported natural GADs exhibit their higher catalytic capacity under acidic conditions, and the crystal structure of *E. coli* GAD revealed the molecular mechanism to some extent [7–9]. The optimal catalysis pH values of bacterial GADs were reported to be in the range from 3.8 to 4.6 [10]. Obviously, this catalytic property of GAD was adverse to the industrial scale bio-manufacture of GABA on account of the cost of desalination process. There are numerous genetically engineered microorganisms reported to possess higher activity in basic conditions and enhanced GABA yield [11]. To date, GABA was mainly synthesized from MSG or L-Glu by applying the purified GAD enzyme or whole-cell/resting-cell biocatalysts [10]. The whole-cell bioconversion exhibits obvious superiority to the catalysis with purified enzyme because of its great efficiency, relatively easy preparation and low costs. Thus, it has been widely applied in industrial scale production of valuable compounds [12]. GAD has also been used in the early diagnosis of type I diabetes. GADs have been isolated from various microbial sources for industrial purposes, such as *E. coli,* lactic acid bacteria (LAB), *Streptococcus salivarius, Bacillus megaterium, Pyrococcus horikoshii, Aspergillus oryzae* and *Neurospora crassa* [4].

*Streptomyces* is rich in natural products with various biological activities, such as antibacterial, antiviral, anti-cholesterol, antiprotozoan and antitumor properties [13]. As a result, *Streptomyces* is well studied for natural product discovery and biosynthesis. However, GADs from this genus have never characterized. Therefore, it is of interest to explore this untapped source for new GADs and reveal their potential in GABA production. In this work, we sequenced and analyzed the genomes of three *Streptomyces* species including *Streptomyces toxytricini* NRRL 15443, *Streptomyces* sp*.* MJ654-NF4 and *Streptomyces chromofuscus* ATCC 49982. Each strain was found to harbor a putative *gad* gene, including *Stgad* from *S. toxytricini*, *Ssgad* from *Streptomyces* sp. and *Scgad* from *S. chromofuscus*. We cloned these three *gad* genes from the corresponding hosts and expressed them in *E. coli* BL21(DE3). Recombinant StGAD, SsGAD and ScGAD were purified using Ni-NTA chromatography for enzymatic studies. These enzymes were functionally characterized as L-glutamate decarboxylase and their enzymatic properties were also investigated. SsGAD and ScGAD showed higher efficiency than previously reported GADs, with *k*_*cat*_*/*K_m_ values of 0.62 and 1.21 mM^− 1^·s^− 1^, respectively. Finally, we tested the capability of engineered *E. coli* BL21(DE3) strains harboring StGAD, SsGAD and ScGAD for GABA production. *E. coli*/SsGAD showed the best performance as whole-cell biocatalyst and gave an accumulated GABA yield of 2.771 kg/L after repeated use for 10 times. This work provides an efficient biosynthetic platform for industrial production of GABA.

## Results

### Amino acid sequence analysis of three putative GADs from *Streptomyces*

**Fig. 2:**
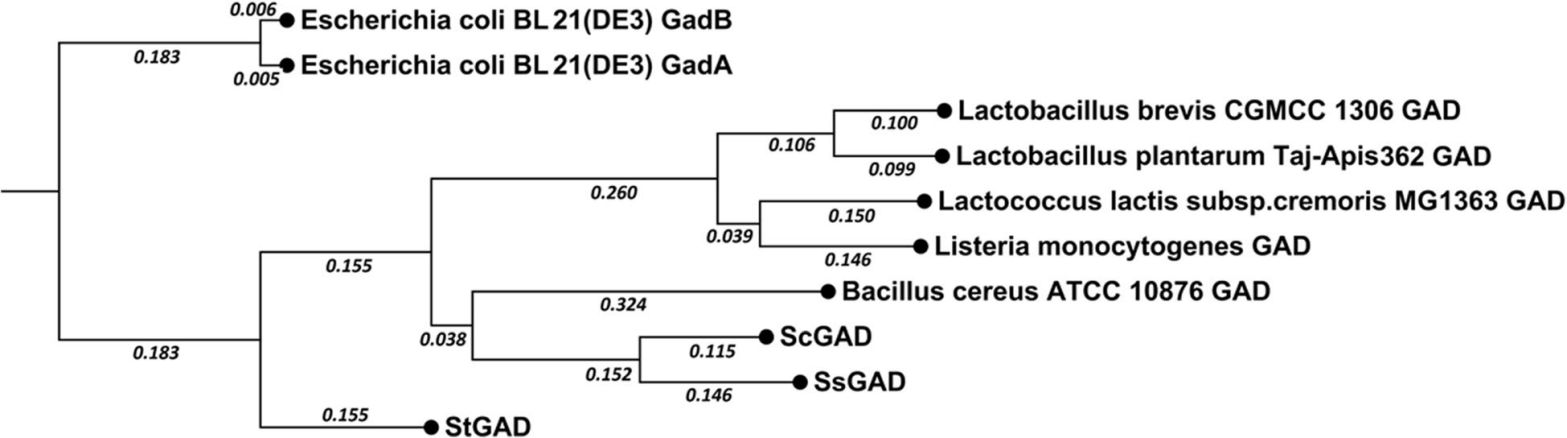
Phylogenetic tree of GADs. The GAD sequences used include StGAD, ScGAD, SsGAD, *Lactobacillus brevis* CGMCC 1306 GAD, *Lactobacillus plantarum* Taj-Apis362 GAD, *Listeria monocytogenes* GAD, *Lactococcus lactis* subsp. *cremoris* MG1363 GAD, *Bacillus cereus* ATCC 10876 GAD, *Escherichia coli* BL21(DE3) GadB, and *Escherichia coli* BL21(DE3) GadA

We have sequenced the genomes of *S. toxytricini* NRRL 15443, *Streptomyces sp.* MJ654-NF4, and *S. chromofuscus* ATCC 49982 [14, 15]. Analysis of these genomes revealed that each of these three *Streptomyces* strains contains a putative GAD gene. Based on the Latin names of the strains, these putative genes were named *Stgad*, *Ssgad* and *Scgad*, respectively. BLAST analysis of the amino acid sequences of these GADs indicated that they shared 53% or lower identities with previously reported GADs listed in Table 1. All these three putative GADs contain the amino acid residues GI(V/S) TY(F)D(T)G (245–250, numbering according to the sequence of ScGAD) conserved for the catalytic site of GADs [16, 17] in Additional file 1: Figure S1. The amino acid residues HV(I)DG(A) ASGG (276–283) are highly conserved in PLP-dependent decarboxylases, such as the GAD from *Lactobacillus brevis* CGMCC 1306 [18, 19]. In addition, the IN(S) T(V/A)SGHKYGLV(A)YPGVGWV(A)L(V/I)WR (307–327) motif is a PLP binding domain, in which the conserved lysine residue (K313) was indicated to be an active site for binding of PLP [16, 19]. Moreover, this lysine residue is essential for GAD decarboxylation, formation of aldimine, hydrolysis and product release. Therefore, the sequence analysis suggested that StGAD, ScGAD and SsGAD are functional GADs. A phylogenetic tree was built for the three *Streptomyces* GADs and several reported GADs, including *Lactobacillus brevis* CGMCC 1306 GAD [20], *Lactobacillus plantarum* Taj-Apis362 GAD [21], *Listeria monocytogenes* GAD [22], *Lactococcus lactis* subsp.*cremoris* MG1363 GAD [23], *Bacillus cereus* ATCC 10876 GAD [24], *Escherichia coli* BL21(DE3) GadB [25], and *Escherichia coli* BL21(DE3) GadA [25]. As shown in Fig. 2, all three *Streptomyces* GADs form their own clades that are different from those reported GADs. StGAD is in one clade, while SsGAD and ScGAD are relatively similar and were grouped into another clade.

**Table 1:** Identities of StGAD, SsGAD and ScGAD with known GADs

Known GADs	*Streptomyces* GADs			References
	StGAD	SsGAD	ScGAD	
	Identities			
*Escherichia coli* BL21(DE3) GadB	50%	48%	46%	[25]
*Escherichia coli* BL21(DE3) GadA	50%	48%	46%	[25]
*Bacillus cereus* ATCC 10876 GAD	52%	52%	53%	[24]
*Listeria monocytogenes* GAD	48%	46%	45%	[22]
*Lactobacillus brevis* CGMCC 1306 GAD	48%	44%	46%	[20]
*Lactococcus lactis* subsp.*cremoris* MG1363 GAD	45%	45%	47%	[23]
*Lactobacillus plantarum* Taj-Apis362 GAD	47%	44%	48%	[21]

### Overexpression and purification of recombinant StGAD, SsGAD, ScGAD and GadB

**Fig. 3:**
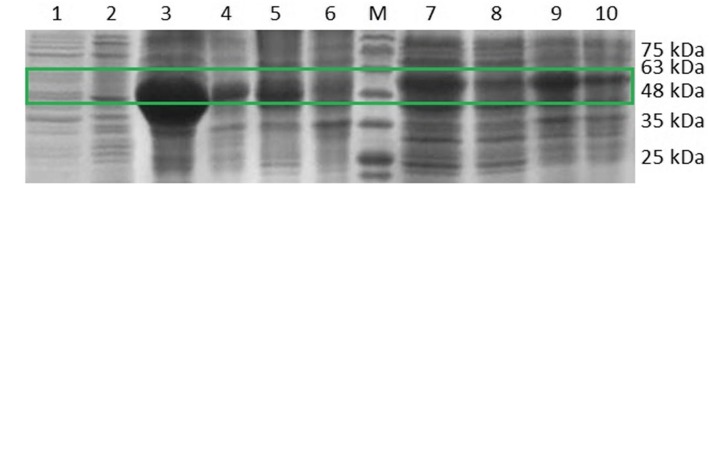
SDS-PAGE analysis of expression and purification of GADs from *E. coli* BL21(DE3). **a** Expression of GadB, StGAD, ScGAD, and SsGAD in *E. coli* BL21(DE3). M: protein ladder; 1 and 2: insoluble and soluble fractions of *E. coli* BL21(DE3)/pET28a (vector control); 3 and 4: soluble and insoluble fractions of *E. coli* BL21(DE3)/GadB; 5 and 6: soluble and insoluble fractions of *E. coli* BL21(DE3)/StGAD; 7 and 8: soluble and insoluble fractions of *E. coli* BL21(DE3)/SsGAD; 9 and 10: soluble and insoluble fractions of *E. coli* BL21(DE3)/ScGAD. **b** GADs purified from the engineered *E. coli* BL21(DE3) strains. 1: GadB; 2: StGAD; 3: SsGAD; 4: ScGAD. StGAD, SsGAD and GadB were expressed in *E. coli* BL21(DE3) at 28 °C, while ScGAD was expressed at 18 °C. The target band of the GADs are in the green box

In order to functionally characterize these putative GADs, we next attempted to express these enzymes in *E. coli* BL21(DE3). GadB is a well characterized GAD from *E. coli* and thus was used as a reference enzyme for comparison. These genes were cloned from the genome of the hosts and ligated into pET28a. The resulting plasmids, including pHW1 (pET28a-GadB), pHW4 (pET28a-StGAD), pHY1 (pET28a-ScGAD) and pHY6 (pET28a-SsGAD), were expressed in *E. coli* BL21(DE3). SDS-PAGE analysis (Fig. 3a) showed that all proteins (52–56 kDa) can be expressed in this strain. The calculated molecular weights were 53 kDa for StGAD, 54.7 kDa for SsGAD, 56.3 kDa for ScGAD and 52.7 kDa for GadB. Notably, StGAD, SsGAD and GadB were expressed well at 28 °C, while more soluble ScGAD was produced in *E. coli* at 18 °C than 28 °C. These four His_6_-tagged GADs were purified by Ni-NTA affinity chromatography to homogeneity (Fig. 3b) for the following enzymatic studies. The isolation yields of GadB, StGAD, SsGAD and ScGAD were 60.5 ± 2.5, 33.1 ± 1.7, 50.0 ± 3.2 and 20.5 ± 2.4 mg/L, respectively.

### Functional identification of three *Streptomyces* GADs

**Fig. 4:**
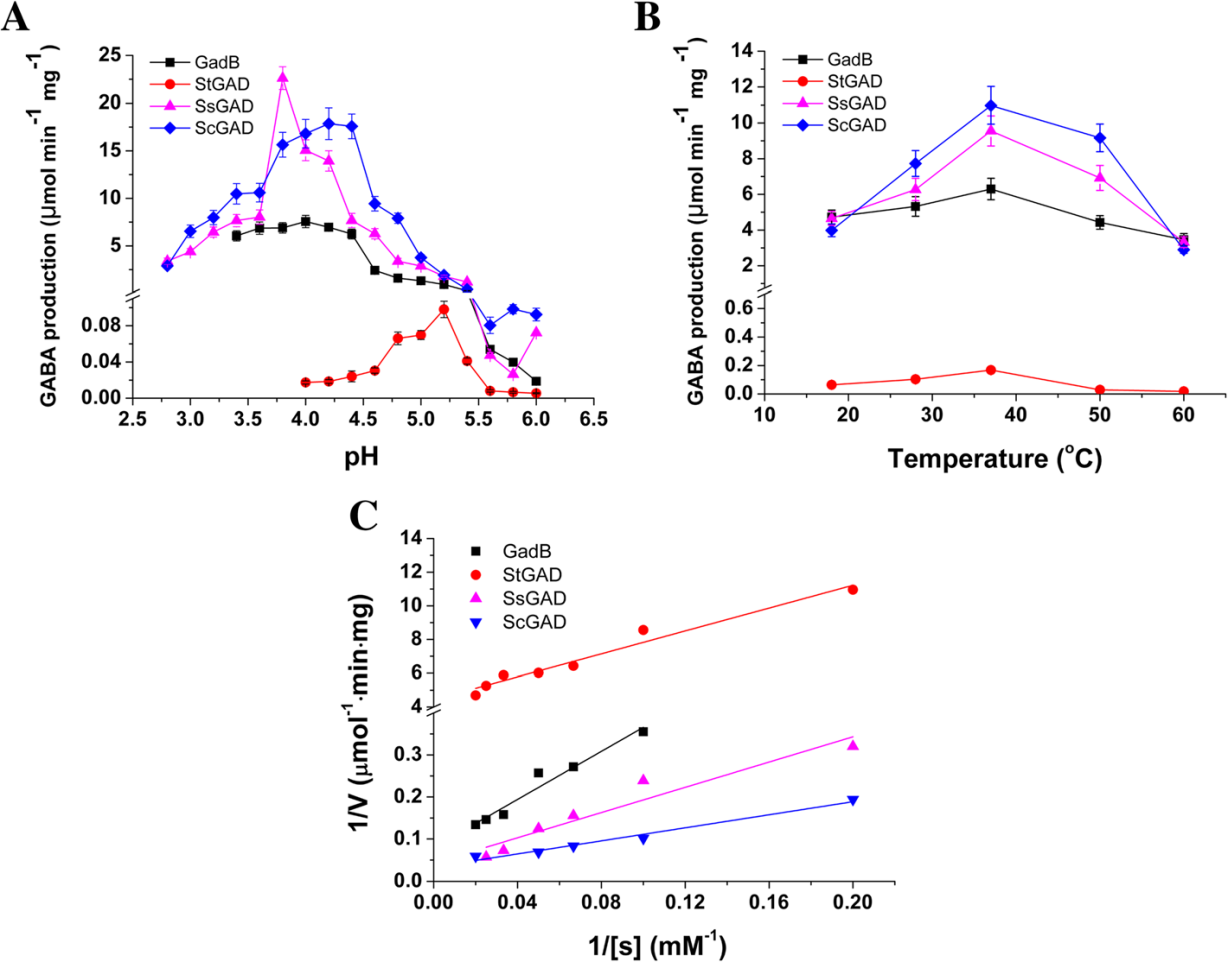
Enzymatic properties of StGAD, SsGAD, ScGAD and GadB. **a** Effect of the reaction pH on GAD activity; **b** Effect of the reaction temperature on GAD activity; **c** Determination of the kinetic parameters via the Lineweaver-Burk plot

The purified GADs were subjected to in vitro reactions using monosodium glutamate (MSG) as the substrate, with GadB from *E. coli* as the positive control. All the three GADs were found to be able to convert MSG to GABA, confirming that they are indeed L-glutamate decarboxylases. GAD requires proton participation to perform the glutamate decarboxylation reaction [26]. The optimum pH of bacterial GADs has been reported to be in the range of 4.0–5.0 [19, 27, 28]. The activity of the three *Streptomyces* GADs were tested at 37 °C and different pH values ranging from 2.6 to 6.0. The optimum activity of StGAD, SsGAD, and ScGAD was observed at pH 5.2, 3.8, and 4.2, respectively (Fig. 4a). These were different from the characterized GadB for which the optimum pH value is 4 [27].

We then tested the activity of StGAD, SsGAD, and ScGAD at five different reaction temperatures including 18, 28, 37, 50 and 60 °C. Interestingly, the optimal temperature for all three GADs were found to be 37 °C (Fig. 4b). The three purified GADs exhibited significantly different catalytic efficiency (*p* < 0.05). ScGAD exhibited the highest conversion rate, which was 68-fold and 1.7-fold of those of StGAD and GadB, respectively. Similarly, SsGAD showed around 60-fold and 1.5-fold stronger catalytic activity than those of StGAD and GadB.

### Determination of the kinetic parameters of the three *Streptomyces* GADs

To understand the properties and catalytic efficiency of the three *Streptomyces* GADs, the K_m,_ *V*_*max*_ and *k*_*cat*_ values of StGAD, SsGAD, ScGAD and GadB were measured using MSG as the substrate and were calculated from corresponding Lineweaver-Burk plots in Fig. 4c. The K_m_ values for StGAD, SsGAD ScGAD and GadB were 7.67 ± 0.55, 35.17 ± 2.12, 23.25 ± 2.07, and 35.83 ± 2.40 mM, respectively (Table 2), indicating that these enzymes have different affinity to MSG. The *V*_*max*_ values for StGAD, SsGAD and ScGAD were determined to be 0.226 ± 0.01, 23.42 ± 1.57 and 29.94 ± 2.16 μmol·min^− 1^·mg^− 1^, respectively. Among the three *Streptomyces* GADs, ScGAD was found to possess the highest efficiency, with a *k*_*cat*_/K_m_ value of 1.21 ± 0.09 mM^− 1^·s^− 1^. SsGAD showed a *k*_*cat*_/K_m_ value of 0.62 ± 0.04 mM^− 1^·s^− 1^, whereas StGAD is the least efficient, with a low *k*_*cat*_/K_m_ of 0.026 ± 0.003 mM^− 1^·s ^− 1^ (Table 2). The *k*_*cat*_/K_m_ values of SsGAD and ScGAD are much higher than GadB, which is 0.31 ± 0.02 mM^− 1^·s ^− 1^. Therefore, SsGAD and ScGAD are more efficient than those previously reported GADs [20, 28, 29].

**Table 2:** Kinetic parameters for StGAD, SsGAD, ScGAD and GadB

Kinetic parameters	*Streptomyces* GADs			GadB
	StGAD	SsGAD	ScGAD	
*V*_*max*_ (μmol·min^− 1^·mg^− 1^)	0.226 ± 0.01^d^	23.42 ± 1.57^b^	29.94 ± 2.16^a^	12.53 ± 0.72^c^
K_m_ (mM)	7.67 ± 0.55^c^	35.17 ± 2.12^a^	23.25 ± 2.07^b^	35.83 ± 2.40^a^
*k*_*cat*_ (s^− 1^)	0.20 ± 0.02^d^	21.96 ± 2.08^b^	28.08 ± 1.99^a^	11.00 ± 0.88^c^
*k*_*cat*_/K_m_ (mM^− 1^·s^− 1^)	0.026 ± 0.003^d^	0.62 ± 0.04^b^	1.21 ± 0.09^a^	0.31 ± 0.02^c^

Data are shown as the mean ± standard deviation (*n* = 3). The different letters indicate significant differences among samples at a significant level of 0.05 (*p* < 0.05), and the same letters indicate no significant differences

### Bioconversion of MSG and L-Glu into GABA using engineered *E. coli* as a whole-cell biocatalyst

**Fig. 5:**
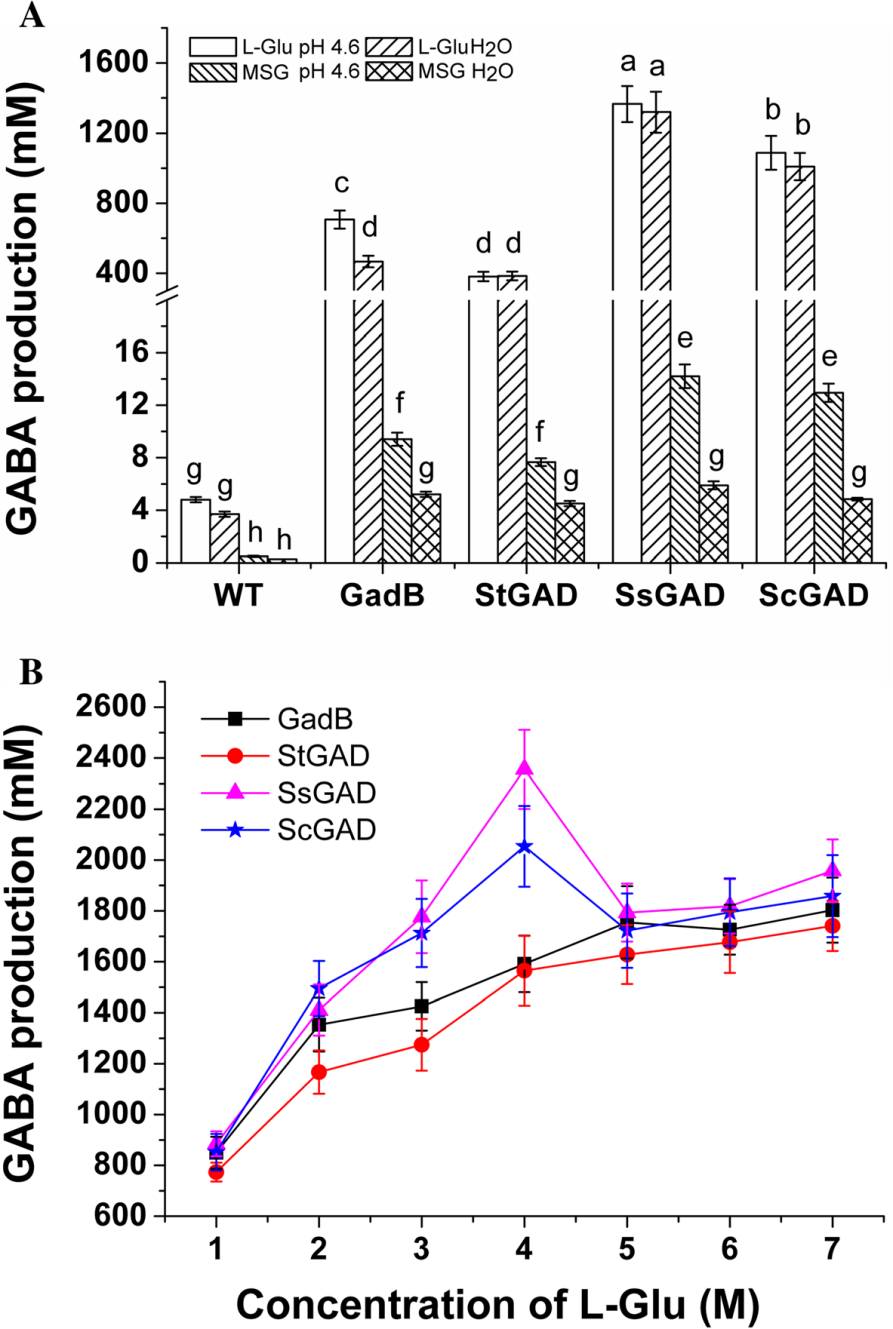
GABA production by whole-cell biocatalysis with enigneered *E. coli* strains. (**a**) Bioconversion of 2 M MSG/L-Glu to GABA in the sodium acetate buffer (pH 4.6) and water; (**b**) Effect of L-Glu concentration on GABA production. Conversions were performed at 37 °C for 1 h with a cell density of OD_600_ 20 and PLP concentratin of 0.2 mM in water. Data are shown as the mean ± standard deviation (n = 3). The different letters indicate significant differences among samples at a significant level of 0.05 (p < 0.05), and the same letters indicate no significant differences

MSG and L-glutamic acid (L-Glu) were respectively used as substrates to generate GABA. The engineered *E. coli* strain harboring StGAD, SsGAD or ScGAD was utilized as a whole-cell biocatalyst for GABA production. *E. coli* BL21(DE3)/GadB was used for comparison and wild type *E. coli* BL21(DE3) (WT) was used as the control. Previous studies reported that *E. coli* GAD showed its full activity at about pH 4–5 [1, 30]. Therefore, we conducted the whole-cell biotransformation reactions in 0.1 M sodium acetate buffer at pH 4.6. We also tested the same reactions in water only. The OD_600_ value of the cells in the biotransformation system was 20. Conversion of 2 M MSG or L-Glu was conducted at 37 °C for 1 h. As shown in Fig. 5a, the four engineered *E. coli* strains had exponentially higher yields of GABA than the wild type. Among these engineering strains, *E. coli* BL21(DE3)/SsGAD showed the best performance, and the yields of GABA from L-Glu reached 1366 ± 102 mM in the sodium acetate buffer and 1319 ± 116 mM in water in one single-batch reaction (Fig. 5a). However, when MSG was used as the substrate, even for *E. coli* BL21(DE3)/SsGAD as the best catalyst, only 14.2 ± 1.0 mM and 5.9 ± 0.3 mM GABA was obtained in the sodium acetate buffer and in water. It was observed that L-Glu was preferred by all these GAD-harboring *E. coli* strains, and the yields from L-Glu were much higher than those from MSG (*p* < 0.05) in both the sodium acetate buffer and water. For example, the yield of GABA by *E. coli*/SsGAD from L-Glu was 170-fold higher than that of MSG. Moreover, there was almost no significant difference between GABA production in the sodium acetate buffer and water when L-Glu was taken as the substrate (Fig. 5a). Therefore, we used L-Glu as the substrate and water as the reaction system for the following whole-cell bioconversion studies.

*E. coli* BL21(DE3)/StGAD, *E. coli* BL21(DE3)/SsGAD, *E. coli* BL21(DE3)/ScGAD, and *E. coli* BL21(DE3)/GadB were then reacted with different concentrations of L-Glu (1–7 M) in water. When the substrate concentration was lower than 4 M, the GABA production efficiency was high for all strains, with yields of 1.5–2.4 M and molar conversion rates of 77.4–88.5% within 1 h (Fig. 5b). However, when the L-Glu concentration was higher, the conversion efficiency decreased. When 7 M of L-Glu was added, the conversion ratio was reduced to 24.9–28.0 mol%. The reaction mixture system cannot be mixed well with shaking due to large amounts of insoluble L-Glu in the system, which might have impacted the reaction efficiency. Additionally, high concentrations of GABA might also inhibit the reaction. Overall, the recombinant *E. coli* strain harboring SsGAD exhibited a conversion capability superior to StGAD, ScGAD and GadB (Figs. 5a and b). Thus, this strain was chosen for the following whole-cell bioconversion studies.

### Effects of cell and PLP concentrations on GABA production

**Fig. 6:**
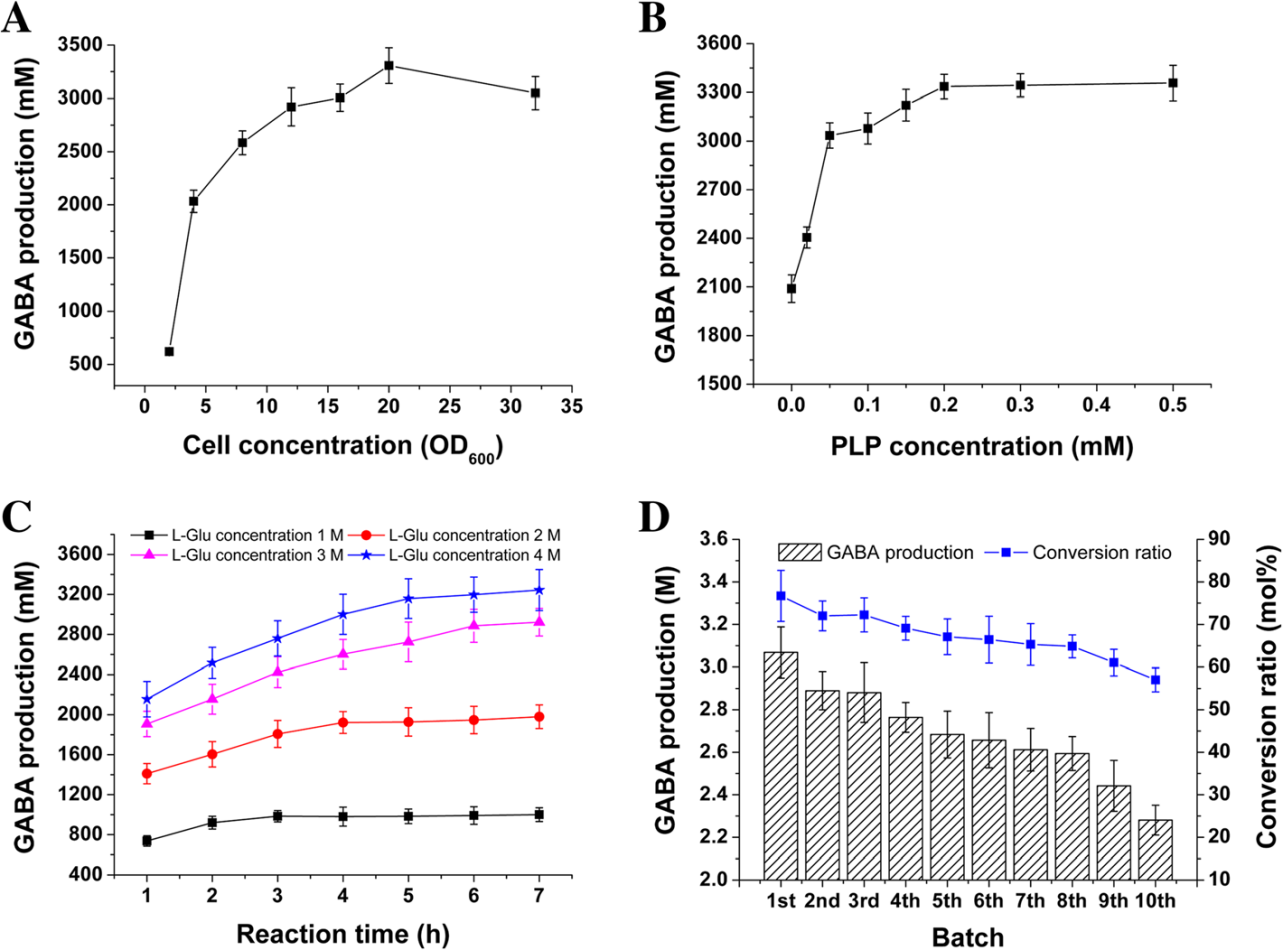
Optimization of GABA production by *E. coli* BL21(DE3)/SsGAD from L-Glu. **a** Effect of the cell concentration on GABA yield. The reactions were performed with 7 M L-Glu in water at 37 °C for 2 h and with a cell density of OD_600_ 20; **b** Effect of the PLP concentration on GABA yield. The reaction was carried out at 37 °C for 2 h with 4 M L-Glu and a cell density of OD_600_ 20 in water. **c** Time course analysis of GABA formation in single-batch reactions with 1 M, 2 M, 3 M, and 4 M L-Glu. The concentrations of cells and PLP were OD_600_ 20 and 0.2 mM, respectively; **d** Batch reactions with reused *E. coli* BL21(DE3)/SsGAD with a cell density of OD_600_ 20

The effects of cell amount and PLP concentration on GABA yield were evaluated with *E. coli* BL21(DE3)/SsGAD. We first tested the reactions of this strain at different cell concentrations ranging from OD_600_ 2 to 32, with 7 M L-Glu at 37 °C for 2 h (Fig. 6a). The L-Glu concentration was set the highest concentration of 7 M to ensure that the substrate is enough for the high cell concentrations. It was found that the conversion rate increased with higher concentrations of cells. OD_600_ 20 showed the highest conversion rate, with a yield of 3.3 M. Further increase in cell concentration did not yield more product, likely due to a too dense reaction system.

PLP is an essential cofactor for GAD-catalyzed decarboxylation of L-Glu [4]. Various concentrations of PLP were then tested with *E. coli* BL21(DE3)/SsGAD at OD_600_ 20. The reactions were performed at 37 °C for 2 h with 4 M L-Glu. Our results showed that GABA yield reached 3335 ± 75 mM with the enhancement of PLP supplementation up to 0.2 M (Fig. 6b). Further increase in the PLP concentration did not increase the production of GABA. This result agrees with those of GADs from *E. coli* NBRC 3806 and *Lactobacillus brevis* HYE1 [17, 31].

### GABA production of GABA from L-Glu by repeated use of *E. coli* BL21(DE3)/SsGAD

We next tested whether *E. coli* BL21(DE3)/SsGAD can be repeatedly used for GABA production, which can reduce the production costs of GABA. The reaction time was first investigated with different concentrations of L-Glu with the cell concentration being OD_600_ 20. When the concentration of L-Glu was 1 M, it took 2 h to reach 92 mol% conversion. However, it took a longer time to reach the highest conversion rate for 2, 3 and 4 M L-Glu (Fig. 6c). In addition, the velocity in the first hour reached the highest for all the reactions and slowed down after 1 h. At the first reaction hour, GABA was produced at 736 ± 52 mM (73.6 mol%, molar conversion ratio), 1410 ± 101 mM (70.5 mol%), 1907 ± 127 mM (63.6 mol%) and 2155 ± 177 mM (53.9 mol%) from 1, 2, 3 and 4 M L-Glu, respectively. Based on the GABA yield and productivity, 2 M L-Glu supplementation was selected for batch reactions (Fig. 6d).

For batch reactions, a cell density of OD_600_ 20 was used. The initial concentration of L-Glu was 2 M and PLP was supplemented at 0.2 mM. The bioconversion was performed at 37 °C for 1 h before an additional 2 M L-Glu was added into reaction system. The cells were then centrifuged and re-used for the next batch. We found that *E. coli* BL21(DE3)/SsGAD can be used for at least 10 batches with a conversion rate of 57–77 mol% (Fig. 6d). The accumulated yield of GABA in one liter of reaction system was 26.9 mol (equal to 2.771 kg) of GABA from a total of 40 mol (equal to 5.885 kg) of L-Glu. This is higher than previously reported 614.15 g/L [1, 3, 4, 11]. The overall molar conversion rate and productivity were about 67% within 20 h and 138 g/L/h, respectively. Therefore, *E. coli* BL21(DE3)/SsGAD is a promising strain for GABA production.

## Discussion

GAD is a PLP-dependent decarboxylase which specifically converts L-Glu into GABA. GABA attracted large interests in its industrial promise, thus, new GADs with higher decarboxylation efficiency is primary and critical to develop cost-effective GABA biosynthetic process. Although *Streptomyces* strains have been well explored for physiologically active compounds, characterization of GADs from this genus has never been reported. In this work, we identified three new GADs from three different *Streptomyces* strains. The function and catalytic activity of StGAD, SsGAD and ScGAD were tested and confirmed with reactions using pure enzymes or cells expressing these GADs.

PLP is an essential cofactor for the efficient interactions between GAD and substrate [32]. The internal aldimine was formed to link PLP and the active-site residue K279 of *L. brevis* CGMCC 1306 GAD, and they catalyzed the decarboxylation reaction through the formation of this kind of Schiff base. Several other amino acid residues, including Ser126, Ser127, Ser276, Ser321, Cys66, Ile206 and His278 in *L. brevis* CGMCC 1306 GAD, were reported to be critical in the orientation of PLP and promotion of decarboxylation reaction [19]. Hence, PLP seems to be necessary for decarboxylation reactions of GADs in vitro. However, for whole-cell biotransformation, PLP is not required because of its natural occurrence in *E. coli* cells. In this work, the GABA yield was increased by 45% with 0.05 mM PLP addition compared to that of without PLP addition (Fig. 6b). When the amount of PLP was over 0.2 mM, GABA production did not increase further. In addition, PLP biosynthetic genes, *pdxS* and *pdxT* from *Bacillus subtilis,* have been reported to be successfully introduced into lysine decarboxylase-overexpressing *E. coli* strain BL-CadA without exogenous PLP requirement [33]. The crystal structure of *Lactobacillus brevis* CGMCC 1306 GAD indicated that a putative substrate pocket containing Lys 279, Thr 64, Thr 205, Phe 65, Phe 334, Cys 66, Gln 166, and Ser 321 and a flexible loop including residues YLGGE (308–312) played the critical role on decarboxylation of L-Glu. The amino acid residue differences among StGAD, SsGAD, and ScGAD could contribute to their significant variations on K_m_ and *k*_*cat*_/K_m_ shown in Table 2.

MSG was utilized as the substrate to determine the kinetic parameters of GadB, StGAD, SsGAD and ScGAD in the in vitro reactions because of its high water solubility (~ 740 g/L). In terms of GABA production with whole-cell biotransformation, reactions with MSG and L-Glu as the substrate exhibited significantly different productivity, which was attributed to GAD’s acidic pH-dependent property. Microbial origin GADs could become inactive at pH values above 6 due to conformational changes [34]. MSG is basic in water and L-Glu is acidic. The pH value was gradually increased (5.6 ± 0.2/6.88 ± 0.1, 5.9 ± 0.1/6.98 ± 0.2, 6.1 ± 0.2/7.09 ± 0.1, and 6.3 ± 0.3/7.26 ± 0.3) when MSG was supplemented into 0.1 M of sodium acetate buffer (pH 4.6)/water at 1 M, 2 M, 3 M and 4 M because of the alpha-amino group, respectively, which makes a disadvantageous pH environment for the catalytic activity of GADs [35]. Accordingly, MSG is not a suitable substrate for GABA production using whole cells. High concentration of MSG in the reaction system increases the pH and osmotic pressure, which have harmful impacts on the decarboxylation reaction and the cells, respectively. Alternatively, L-Glu is a better substrate to biosynthesize GABA on account of maintaining the acidic pH condition, as shown in some previous studies [3, 11, 31]. Thus, in this study, we used L-Glu as the substrate to achieve a favorable acidic pH environment for GABA production. L-Glu has much lower solubility in water than MSG, around 7.5 g per liter of water at 20 °C. As a result, the majority of L-Glu in the reaction system was in solid form and it can be continuously dissolved into water as the conversion proceeds [12]. Correspondingly, much higher yields of GABA were obtained from L-Glu than MSG (Fig. 5a), supporting that L-Glu is a better substrate for GABA production using whole cells.

The whole-cell or resting cell biocatalysis has a lot of advantages over in vitro enzymatic reactions because of the simple production process, high efficiency and low costs. Although ScGAD showed a higher *k*_*cat*_/K_m_ value than SsGAD (1.21 vs 0.62 mM^− 1^·s^− 1^) (Table 2), *E. coli* BL21(DE3)/SsGAD performed better than the strain harboring ScGAD. This is likely due to the lower expression level of ScGAD than SsGAD (20.5 vs 50 mg/L) in *E. coli*. The resting cells of *E. coli*/GADs could be reused unless they are disrupted, resulting in the release of intracellular GAD out of cells and inactivation/degradation. In our work, the engineered *E. coli* cells with SsGAD is a promising candidate for an economically viable industrial scale production of GABA.

## Conclusions

In conclusion, we discovered three putative GAD genes from the genomes of *S. toxytricini* NRRL 15443, *Streptomyces sp.* MJ654-NF4, and *S. chromofuscus* ATCC 49982. These GADs, including StGAD, SsGAD, and ScGAD, were cloned and heterologously expressed in *E. coli* BL21(DE3). The functions of these enzymes were characterized. StGAD, SsGAD, and ScGAD showed different enzymatic characteristics, and the catalytic efficiencies were different among the GADs from different *Streptomyces* strains. The *k*_*cat*_/K_m_ values of SsGAD and ScGAD are higher than previously reported GADs. An efficient whole-cell biocatalyst was developed from SsGAD to produce GABA in a cost-effective manner. In view of GABA production, engineered *E. coli* BL21(DE3)/SsGAD cells could be used for at least ten batches with an overall conversion rate of 67%. The accumulated GABA yield reached 2.771 kg from 5.885 kg L-Glu in one liter of reaction system. Thus, this engineered strain has potential applications for industrial production of GABA as a highly efficient biocatalyst.

## Methods

### Strains, plasmids and media

*E. coli* XL1-Blue was used for general cloning purposes. *E. coli* BL21(DE3) was used for GAD expression and GABA production. The pJET1.2 and pET28a (+) vectors were used for cloning and expression, respectively. Phusion DNA polymerase, restriction enzymes and T4 DNA ligase were purchased from New England Biolabs. All primers were synthesized by Sigma-Aldrich. *E. coli* strains were routinely cultivated in Luria-Bertani (LB) medium (Fisher Scientific, USA) at 37 °C. Carbenicillin and kanamycin were used at 50 μg/mL as needed for selection of correct clones. All chemicals were of analytical grade and purchased from Fisher Scientific.

### Cloning of *gad* genes from *Streptomyces* and plasmid construction

Three *gad* genes from *S. toxytricini* NRRL 15443 (StGAD, GenBank accession number MK303594), *Streptomyces sp.* MJ654-NF4 (SsGAD, GenBank accession number MK303595), *S. chromofuscus* ATCC 49982 (ScGAD, GenBank accession number MK303596) and *gadB* from *E. coli* BL21(DE3) (GenBank accession number ACT43333) were PCR amplified using primers listed in Table 3. The PCR reactions were performed using the following touchdown conditions: initial denaturation at 98 °C for 5 min, followed by 20 cycles of denaturation (98 °C for 30 s), annealing (75 °C with 0.5 °C decrease in each cycle for 30 s), and elongation (72 °C for 2 min), then followed by 20 additional cycles of denaturation (98 °C for 30 s), annealing (65 °C for 30 s), and elongation (72 °C for 2 min), with a final extension at 72 °C for 10 min. The resultant 1395-bp *Stgad*, 1401-bp *Ssgad*, 1437-bp *Scgad* and 1401-bp *gadB* products were ligated into the pJET1.2 cloning vector, yielding pHW3, pHY15, pHY10 and pHW2 (Table 4). The first three plasmids were digested with NdeI and HindIII, and pHY2 that contains *gadB* was digested with NdeI and XhoI for verification. These *gad* genes were excised from the pJET1.2-derived plasmids and ligated into the pET28a (+) vector to yield pHW4, pHY6, pHY1 and pHW1 (Table 4), respectively. To confirm the sequences, these pET28a (+)-derived plasmids were then sent out for sequencing using the Sanger method.

**Table 3:** Primers for amplifying the GAD genes from *E. coli* and *Streptomyces*

Gene	Primers	Restriction site
*gadB*	Forward: 5′-CGCCATATGGATAAGAAGCAAGTAACG-3′	*Nde*I
	Reverse: 5′-CCCTCGAGTCAGGTATGTTTAAAGCTGTT-3′	*Xho*I
*Stgad*	Forward: 5′-AACATATGGCTCTCCACAAGACGAAGGA-3′	*Nde*I
	Reverse: 5′-AAAAGCTTTTAGTGGTGGAAGCCGGAGCGGGGA-3′	*Hind*III
*Ssgad*	Forward: 5′-AACATATGGCCTTGTACAAGGGCACCG-3′	*Nde*I
	Reverse: 5′-AAAAGCTTTTAGTGGTGGAAGCCGGCGCGGACC-3′	*Hind*III
*Scgad*	Forward: 5′-AACATATGCCACTCCACCAAGGCGCGGACA-3′	*Nde*I
	Reverse: 5′-AAAGCTTTTAGTGGTGGAAGGCGGTGGCGGCC-3′	*Hind*III

The restriction sites are underlined

**Table 4:** List of plasmids used in this work

Plasmids	Description	Source
pHW2	*gadB* in pJET1.2	This Work
pHW1	*gadB* in pET28a	This Work
pHW3	*Stgad* in pJET1.2	This Work
pHW4	*Stgad* in pET28a	This Work
pHY10	*Scgad* in pJET1.2	This Work
pHY1	*Scgad* in pET28a	This Work
pHY15	*Ssgad* in pJET1.2	This Work
pHY6	*Ssgad* in pET28a	This Work

### Expression of StGAD, SsGAD, ScGAD and GadB in *E. coli* BL21(DE3) and enzyme purification

*E. coli* BL21(DE3) harboring pHW4, pHY6, pHY1 and pHW1 were grown at 37 °C with shaking at 250 rpm in 3 mL of LB medium containing kanamycin (50 μg/mL) for about 12 h. The seed cultures were respectively transferred to 100 mL of LB broth containing kanamycin (50 μg/mL) with shaking at 250 rpm at 37 °C until the OD_600_ value reached 0.4–0.6. Isopropyl β-D-1-thiogalactopyranoside (IPTG) was added at a final concentration of 200 μM to induce GAD expression. The cultures were incubated for an additional 16 h at 28 °C (pHW4, pHY6, pHW1) or 18 °C (pHY1) with shaking at 250 rpm before harvest. After centrifugation at 12,000×g for 10 min at 4 °C, the cell pellets were re-suspended in the lysis buffer (20 mM Tris–HCl, 0.5 M NaCl, pH 7.9) with 1 mM dithiothreitol and disrupted by ultrasonication (Misonix Sonicator 3000, Misonix Inc., USA) on ice. The cell lysates were centrifuged at 12,000×g for 10 min. The supernatants were collected and analyzed by SDS-PAGE as soluble fractions. Cell debris were dissolved in 8 M urea solution and analyzed on SDS-PAGE as insoluble fractions.

To purify recombinant StGAD, SsGAD, ScGAD, and GadB, the supernatants from the cell lysates were loaded on a HisPur™ Ni-NTA affinity column (Thermo Scientific, Rockford, USA). After washing column with cold wash buffer (50 mM Tris-Hcl, 2 mM EDTA, 20 mM imidazole, pH 7.9), the recombinant StGAD, SsGAD, ScGAD, and GadB proteins were finally eluted with elution buffer (50 mM Tris-Hcl, 2 mM EDTA, 250 mM imidazole, pH 7.9). The fractions were concentrated and desalted using the 30 K Macrosep Advance Centrifugal Device (Pall Corporation, New York, USA). The protein concentrations were determined using the Bradford assay [36]. These enzymes were stored in 50% glycerol (*v*/v) at − 20 °C.

### Sodium dodecyl sulfate-polyacrylamide gel electrophoresis (SDS-PAGE) analysis of protein expression

SDS-PAGE was performed using 12% separation gel, 4% stacking gel, and Laemmli’s Tris-glycine electrolyte buffer system at pH 8.3 on a discontinuous vertical slab electrophoresis system [6]. The standard marker was the BLUEstain™ protein ladder obtained from Gold Biotechnology Inc. (St. Louis, MO, USA) with a molecular weight range of 11–245 kDa. After electrophoresis, the gel was stained with 0.1% Coomassie brilliant blue R-250 and destained with 12–15% acetic acid in water (v/v).

### Enzymatic activity assay and quantification of GABA formation

The activities of StGAD, SsGAD, ScGAD and GadB were examined through in vitro reactions based on the Berthelot method with some modifications [3, 37]. The 2.0-mL reaction mixture consisted of 200 mM Na_2_HPO_4_-citric acid buffer (pH 5.2 for StGAD, pH 3.8 for SsGAD, pH 4.2 for ScGAD and pH 4.0 for GadB), 50 mM L-MSG, 0.01 mM PLP, and 50–100 μL of purified enzyme. The mixtures were thoroughly mixed and incubated at 37 °C for 30 min and then inactivated by boiling for 5 min. The reaction mixtures were centrifuged, and supernatants were collected for measurement of GABA using the Berthelot reaction method. The Berthelot reaction was carried out with a total volume of 2.5 mL which was composed of 1.0 mL of reaction sample, 500 μL of H_2_O, 100 μL of 200 mM sodium borate (pH 9.0), and 500 μL of 6% phenol and 400 μL of 5% (*w*/*v*) sodium hypochlorite. The mixtures were thoroughly mixed, boiled for 10 min, and then immediately placed on ice bath for 20 min. GABA concentration was calculated by measuring the absorbance at 630 nm. 4-Aminobutyric acid (Acros Organics, New Jersey, USA) was used as standard.

### Investigation of reaction conditions and kinetic parameters for the recombinant GADs

#### Optimum pH and temperature assay

The effect of pH on the activity of the recombinant GADs was determined using 200 mM Na_2_HPO_4_-citric acid buffer at various pH values (2.6–6.0) at 37 °C for 30 min. The effect of temperature on GAD activity was measured by incubating the enzymes at the optimum pH value for 30 min at different temperatures (18 °C – 60 °C). Subsequently, the formation of GABA in each reaction was quantified and compared.

#### Determination of kinetic parameters of GADs

The kinetic parameters were estimated through in vitro reactions containing different MSG concentrations from 5 mM to 100 mM, 0.01 mM PLP, 200 mM Na_2_HPO_4_-citric acid buffer with corresponding optimum pH value, and 50–100 μL of purified enzyme. The K_m_ and *V*_*max*_ values were estimated by using double reciprocal via the Lineweaver-Burk plot [17]. The *k*_*cat*_ and *k*_*cat*_/K_m_ were then calculated.

### Whole-cell bioconversion process

After induction for 16 h at 28 °C or 18 °C, wild type *E. coli* BL21(DE3) and the engineered strains harboring StGAD, SsGAD, ScGAD and GadB were collected by centrifugation at 12,000×*g* for 20 min and then resuspended in the 0.1 M sodium acetate buffer at pH 4.6 or water with MSG or L-Glu as the substrate at appropriate concentrations. Cell density was indicated by the OD_600_ value. The reaction mixtures were incubated at 37 °C for the production of GABA.

### Statistical analysis

Assays were conducted in triplicate and values were expressed as the mean ± standard deviation. Thereafter, one-way analysis of variance and subsequent Tukey-Kramer multiple-comparison tests were conducted to evaluate the significance of differences (*p* < 0.05).

## Additional file

Additional file 1: Supplementary data. (DOCX 43 kb)

## Abbreviations

GABA: Gamma(γ)-aminobutyric acid
GAD: L-glutamate decarboxylase
LAB: lactic acid bacteria
L-Glu: L-glutamate or L-glutamic acid
MSG: monosodium glutamate
SDS-PAGE: sodium dodecyl sulfate-polyacrylamide gel electrophoresis
WT: wild type
