# Supplementary material for: Identification of new glutamate decarboxylases from Streptomyces for efficient production of γ-aminobutyric acid in engineered Escherichia coli
Source: J Biol Eng. 2019 Mar 21;13:24. doi: 10.1186/s13036-019-0154-7 (PMC6429771; doi:10.1186/s13036-019-0154-7)
Supplement: Supplementary file 1 — Supplementary data. (DOCX 43 kb) [file 13036_2019_154_MOESM1_ESM.docx]

**Supporting Information**

**for**

**Identification of new glutamate decarboxylases from *Streptomyces* for efficient production of γ-aminobutyric acid in engineered *Escherichia coli***

Haina Yuan^1,2^, Hongbo Wang^1^, Ozkan Fidan^1^, Yong Qin^3^, Gongnian Xiao^2^*, Jixun Zhan^1^*

1 Department of Biological Engineering, Utah State University, 4105 Old Main Hill, Logan, UT 84322-4105, USA

2 School of Biological and Chemical Engineering, Zhejiang Provincial Collaborative Innovation Center of Agricultural Biological Resources Biochemical Manufacturing, Zhejiang Provincial Key Lab for Chem&Bio Processing Technology of Farm Produces, Zhejiang University of Science and Technology, Hangzhou, Zhejiang 310023, China

3 Hangzhou Viablife Biotech Co., Ltd., 1 Jingyi Road, Yuhang District, Hangzhou, Zhejiang 311113, China

**Correspondence:** [jixun.zhan@usu.edu](mailto:jixun.zhan@usu.edu), [xiaogongnian@126.com](mailto:xiaogongnian@126.com)

**Figure S1**. Partial amino acid sequence alignment of StGAD, SsGAD and ScGAD with previously known GADs. The known GADs include GadB (GenBank accession number ACT43333.1) and GadA (GenBank accession number ACT45166.1) from *E. coli* BL21(DE3), a GAD (GenBank accession number ADG02973.1) from *Lactobacillus brevis* CGMCC 1306, a GAD (GenBank accession number AHG59384.1) from *Lactobacillus plantarum* Taj-Apis362, a GAD (GenBank accession number CAL97772.1) from *Lactoccus lactis* subsp. *cremoris* MG1363, a GAD (GenBank accession number KSZ472875.1) from *Listeria monocytogenes*, and a GAD (GenBank accession number KFL74542.1) from *Bacillus cereus* ATCC 10876.
